# In Situ and Invasive Ductal Carcinoma Within a Borderline Phyllodes Tumor

**DOI:** 10.4021/wjon2010.01.1203

**Published:** 2010-02-01

**Authors:** Amel Trabelsi, Soumaya Ben Abdelkrim, Wided Stita, Mohamed Zaher Boudagga, Faten Hammedi, Moncef Mokni

**Affiliations:** a Department of Pathology, Farhat Hached Hospital, Sousse, Tunisia; b Department of Medical Oncology, Farhat Hached Hospital, Sousse, Tunisia

**Keywords:** Phyllodes tumor, Breast carcinoma, Intraductal carcinoma

## Abstract

A rare case of a borderline phyllodes tumor with simultaneous intraductal and infiltrating duct carcinoma is reported. The patient was a 52-year-old woman with a breast tumor detected by physical examination. A simple mastectomy was performed. The excised tumor had a macroscopic appearance of a phyllodes tumor. After histological examination, the diagnosis of ductal carcinoma within a borderline phyllodes tumor was made. Immunohistochemical staining revealed that the epithelial component was positive for Epithelial Membrane Antigen and cytokeratin. No metastasis was detected in the axillary lymph nodes and the patient didn’t receive any adjuvant therapy. No recurrence or metastasis has been observed 38 months after the surgery.

## Introduction

Phyllodes tumors are distinctly uncommon lesions in the female breast. They constitute less than 1% of all breast tumors and 2 - 3% of fibroepithelial breast tumors [1]. Most of them are benign, but up to 30% show malignant stroma [2]. Malignant alteration of epithelial component is extremely rare. This report presents a case of in situ and ductal carcinoma within a borderline phyllodes tumor.

## Case Report

A 52-year-old woman presented with a painful mass in her right breast for the five last months. She had no history of mammary disease. Physical examination revealed a mobile firm tumor, 15 x 15 cm in size, in the outer half of the right breast. The axillary lymph nodes were not palpable. Ultrasound and mammography showed a 20 cm solid and heterogeneous mass with sharp margins. A percutaneous biopsy of the mass showed a fibroepithelial tumor with atypical spindle cells, and simple right mastectomy was performed. Grossly, the excised tumor was 15 x 12 x 6 cm in size and appeared well circumscribed. The cut surface was gray-white with some hemorrhagic and fragile appearing areas. Histological examination showed a biphasic tumor, consisting predominantly of a stromal overgrowth that formed protrusions into cleft-like spaces. The degree of stromal cellularity varied from mild to severe and there was moderate cytologic atypia. Mitotic count was seven per ten high power fields in the most active areas.

**Figure 1:**
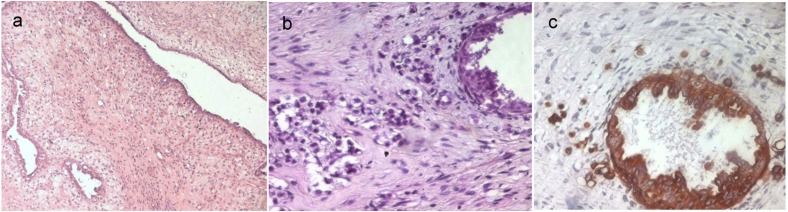
a: A borderline phyllodes tumor (HE x 50); b: The epithelial component of the phyllodes tumor showed foci of in situ and invasive ductal carcinoma (HE x 400); c: Immunoreactivity for cytokeratin of the intraductal and invasive carcinoma (IHC x 400).

These features were consistent with the diagnosis of a borderline phyllodes tumor (Fig. 1a). The epithelial component of this tumor showed foci of ductal carcinoma in situ with a massive and cribriform pattern and foci of invasive mammary carcinoma (Fig. 1b). The ductal carcinoma in situ was of an intermediate nuclear grade. The invasive carcinoma was of no special type, Scarff-Bloom-Richardson (SBR) grade II. There were no oestrogen or progesterone receptors in either the stromal or the epithelial elements. Immunohistochemical stain showed immunoreactivity for Epithelial Membrane Antigen and cytokeratin in the epithelial component (Fig. 1c), while spindle cells were completely negative for these markers.

The patient had a right axillary lymph node dissection, and subsequent pathological examination didn’t reveal node positive for metastatic carcinoma. The patient didn’t receive any adjuvant therapy. Neither recurrence nor metastasis has been detected 38 months after removal of the tumor.

## Discussion

A phyllodes tumor or cystosarcoma phyllodes is an uncommon combined fibroepithelial breast tumor. Its separation from fibroadenoma is based on its greater degree of stromal cellularity. This tumor was first described by Johannes Muler in 1838 [1, 3]. Phyllodes tumors are classified as benign, low-grade malignant (borderline) and malignant lesions. Assessment of malignant potential is based on increased mitotic activity, increased cytologic atypia, stromal overgrowth, increased stromal cellularity and lack of circumscription or ‘pushing’ margins. Heterologous stromal elements can be found in benign, borderline and malignant phyllodes tumors [4]. The epithelial component of phyllodes tumors may show various types of changes like apocrine and squamous metaplasia, adenosis and less commonly proliferative changes [5]. However, carcinoma arising within a phyllodes tumor is rare with less than 30 cases reported in the literature [6]. Most of these cases are of lobular type [7].

The other reported subtypes include in situ and invasive ductal (no special type) carcinoma, tubular carcinoma and squamous cell carcinoma. The stroma is benign in the majority of the cases [2, 6]. The patients' age ranged from 26 to 80 years, with most of them in the 5^th^ or 6^th^ decades, similarly to our patient. The pathogenesis of coexistent carcinoma and phyllodes tumor is controversial. Some authors consider that the disease is due to a sudden transformation of the hyperplastic epithelium of the phyllodes tumor, and others state that the carcinoma is caused haphazardly in the mammary gland adjunct to the phyllodes tumor [7]. In most cases, it is very difficult to preoperatively assess the existence of a carcinoma within a phyllodes tumor. This is due to the fact that the phyllodes tumor usually takes up a larger area than the carcinoma [8]. In our case, the malignant epithelial component was detected after postoperative histologic examination. The prognosis of carcinoma in phyllodes tumor is generally favourable because the stroma is benign in the majority of the cases and cancer detection often occurs early [9]. Lymph node metastases associated with a carcinoma within a phyllodes tumor are extremely rare [2, 6, 7]. Treatment must be performed according to the carcinomatous component independently of the phyllodes tumor [6].

In conclusion, the present case emphasizes the fact that phyllodes tumor may harbor intraductal and invasive carcinoma. Although the stroma in these tumors is commonly the aggressive component, the epithelial component also requires close histological appraisal.
